# Active β-catenin is regulated by the PTEN/PI3 kinase pathway: a role for protein phosphatase PP2A

**DOI:** 10.18632/genesandcancer.128

**Published:** 2016-11

**Authors:** Amit Persad, Geetha Venkateswaran, Li Hao, Maria E. Garcia, Jenny Yoon, Jaskiran Sidhu, Sujata Persad

**Affiliations:** ^1^ Department of Pediatrics, University of Alberta, Edmonton, Alberta, Canada

**Keywords:** ABC, β-Catenin, PP2A, PI3K/PTEN pathway

## Abstract

Dysregulation of Wnt/β-catenin signaling has been associated with the development and progression of many cancers. The stability and subcellular localization of β-catenin, a dual functional protein that plays a role in intracellular adhesion and in regulating gene expression, is tightly regulated. However, little is known about the transcriptionally active form of β-catenin, Active Beta Catenin (ABC), that is unphosphorylated at serine 37 (Ser37) and threonine 41 (Thr41). Elucidating the mechanism by which β-catenin is activated to generate ABC is vital to the development of therapeutic strategies to block β-catenin signaling for cancer treatment. Using melanoma, breast and prostate cancer cell lines, we show that while cellular β-catenin levels are regulated by the Wnt pathway, cellular ABC levels are mainly regulated by the PI3K pathway and are dependent on the phosphatase activity of the protein phosphatase PP2A. Furthermore, we demonstrate that although the PI3K/PTEN pathway does not regulate total β-catenin protein levels within the cell, it plays a role in regulating the subcellular localization of β-catenin. Our results support a novel functional interaction/cross-talk between the PTEN/PI3K and Wnt pathways in the regulation of the subcellular/nuclear levels of ABC, which is crucially important for the protein's activity as a transcription factor and its biological effects in health and disease.

## INTRODUCTION

Beta (β)-catenin is a multifunctional protein that serves a structural role at the adherens junctions [1] and a regulatory function as a transcriptional co-activator of the *wnt/wingless* signal transduction pathway. Activation of the Wnt pathway results in stabilization and accumulation of β-catenin in the cytosol, and its subsequent localization in the nucleus. Under normal conditions, cytosolic levels of β-catenin are regulated by the ubiquitin-proteasome system. Targeted phosphorylation of highly conserved serine (Ser) and threonine (Thr) residues (Ser 33, Ser 37, Thr 41, and Ser 45) at the NH_2_-terminal domain of β-catenin, by glycogen synthase kinase-3 beta (GSK-3β) and Casein Kinase (CK)-1 [1, 2, 3] targets β-catenin for degradation. This phosphorylation event requires association with axin and the tumor suppressor, adenomatous polyposis coli (APC) [4], collectively comprising the destruction complex. In the presence of Wnt signaling, the destruction complex is inactivated resulting in the cytoplasmic accumulation of β-catenin and its subsequent translocation to the nucleus. In the nucleus, β-catenin interacts with the T-cell Factor (TCF)/lymphoid enhancer factor (LEF) family of transcription factors and activates transcription of Wnt target genes such as cyclin D1, VEGF-A and C-Myc [2, 3, 5]. Deregulation of the *Wnt* signaling pathway results in increased β-catenin levels in the nucleus and consequently, the constitutive transcription of *Wnt* target genes [2-4]. Increased transcriptional activity of β-catenin has been associated with the development and progression of many cancers [4, 6].

In 2002, Staal and colleagues demonstrated that β-catenin molecules that were dephosphorylated at residues Ser37 and Thr41 at its N-terminal had enhanced transcriptional activity [7]. This dephosphorylated, transcriptionally active form of β-catenin was referred to as Active Beta Catenin (ABC) [7]. The generation of ABC required the involvement of phosphatases, although the identity of the phosphatase(s) was not established [7]. A putative role for protein phosphatase 2A (PP2A), a diverse heterotrimeric protein phosphatase, as a regulator in the generation of ABC has been suggested [8, 9]. This hypothesis is supported by the observation that the catalytic subunit of PP2A exerts a positive effect and the inhibitory regulatory B56 subunit exerts a negative influence on Wnt/β-catenin signaling in mammalian cells and *Xenopus* embryo explants [10, 11].

While cellular stability of β-catenin is known to be regulated by the canonical Wnt pathway, several mechanisms have been implicated in the regulation of the transcriptional activity of β-catenin. EGF stimulation of cells results in translocation of β-catenin to the nucleus and increases its transactivation function without altering its stability and phosphorylation levels by GSK3β [12]. In addition, the phosphatidylinositol 3 kinase (PI3K) pathway was shown to interact with and modulate canonical Wnt/β-catenin pathway activation via several context-dependent mechanisms [13-17].

An increasing body of evidence suggests that deregulation of the PI3K pathway is strategically used by tumors to attain growth advantage and survival [18]. Interestingly, perturbation of the tumor suppressor PTEN, negative regulator of the PI3K pathway, rivals the frequency of p53 mutations observed in many human cancers [18]. PTEN is mutated or lost in a large proportion of melanomas, glioblastomas, prostate, breast, thyroid and endometrial cancers [19, 20]. Loss of expression or function of PTEN is due not only to mutation and allelic loss [21, 22], but also to functional inactivation by epigenetic silencing [19] or altered subcellular localization [23]. Absence or inactivity of PTEN deregulates the PI3K pathway leading to activation of the downstream effector, protein kinase B (PKB)/AKT via its phosphorylation at two critical amino acids: Ser473 and Thr308. The resulting constitutive activation of PKB/AKT induces a sustained proliferative and anti-apoptotic cellular response.

The Wnt/β-catenin and PI3K/PTEN signaling pathways include GSK3β as a common point of intersection [24]. However, while several studies support GSK3β as the node for crosstalk between these two pathways [25-27], others have suggested that this may not be the case [28, 29].

In this study we used several melanoma, breast and prostate cancer cell lines to understand the mechanism by which ABC is regulated. In light of the frequent deletion or inactivation of the PTEN gene in these cancers as well as the evidence for crosstalk between these pathways, we investigated the regulation of ABC by the Wnt and PI3K pathways. Currently, the involvement of the Wnt/β-catenin and/or PI3K pathways in the generation of ABC is unknown. Our study demonstrates that ABC levels are elevated with PTEN loss/inactivity. Furthermore, our results suggest that while cellular levels of β-catenin are regulated by the canonical Wnt pathway, cellular ABC levels are mainly regulated by the PI3K pathway, and involve the phosphatase activity of PP2A.

## RESULTS

### Increase in cellular levels of β-catenin and ABC is associated with loss of PTEN

**Figure 1:**
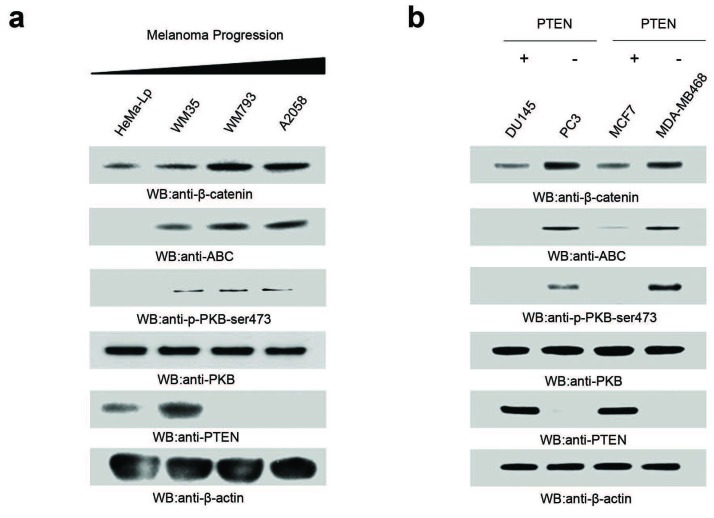
Cellular levels and localization of β-catenin and ABC are altered by loss of PTEN **A.** Western blot analysis of cellular levels of β-Catenin, ABC, PKB-Ser473-P/PKB and PTEN in normal melanocytes (Hema-LP), radial (WM-35) and vertical (WM-793) growth phase, and metastatic (A2058) melanoma cell lines **B.** Western blot analysis of cellular levels of β-Catenin, ABC, PKB-Ser473-P/AKT and PTEN in PTEN expressing and PTEN-null prostate and breast cancer cell lines. Each group of data is representative of 4 or more experiments.

We used a panel of four cell lines representing melanoma progression, including the melanocytic line (HeMa-Lp), radial growth phase (WM35), vertical growth phase (WM793B) and metastatic melanoma (A2058), to assess the cellular levels of total β-catenin and ABC. Total β-catenin was elevated in WM35, WM793B and A2058 cell lines compared to HeMa-Lp cell line (Figure 1A). Similarly, ABC levels were high in WM35, WM793B and A2058 while undetectable in the HeMaLp cells (Figure 1A).

Interestingly, there was a corresponding loss of PTEN expression in the WM793B and A2058 cell lines with high P-PKB Ser473 levels supporting a constitutively active PI3K pathway (Figure 1A). Although the WM35 cells expressed PTEN, they also expressed constitutively elevated levels of P-PKB-Ser-473 suggesting a deregulated PI3K pathway. We have previously demonstrated that WM35 expresses an inactive PTEN [30]. The HeMaLp cells had undetectable levels of P-PKB-Ser-473 indicating a regulated PI3K pathway. Levels of PKB were similar in all cell lines.

Given the inverse relationship between PTEN and β-catenin/ABC expression in the melanoma cell lines, we compared the levels of β-catenin/ABC and PI3K/PKB in PTEN-positive or PTEN-deficient prostate and breast cancer cell lines. β-catenin/ABC levels were substantially higher in the PTEN-null cell lines relative to cell lines with PTEN (Figure 1B).

### Cellular ABC levels decrease upon reintroduction of PTEN-wild-type in PTEN-null A2058 cells

To further investigate the relationship between the PI3K/PTEN pathway and β-catenin/ABC, A2058 metastatic melanoma cells were transiently transfected with pEGFP-PTEN-wild-type (pEGFP-PTEN). Total cellular β-catenin and ABC were evaluated by Western Blot and immunofluorescence (IF) analysis and levels of β-catenin phosphorylated at Ser33, Ser37, Thr41 or Ser45 were evaluated by Western Blot.

**Figure 2:**
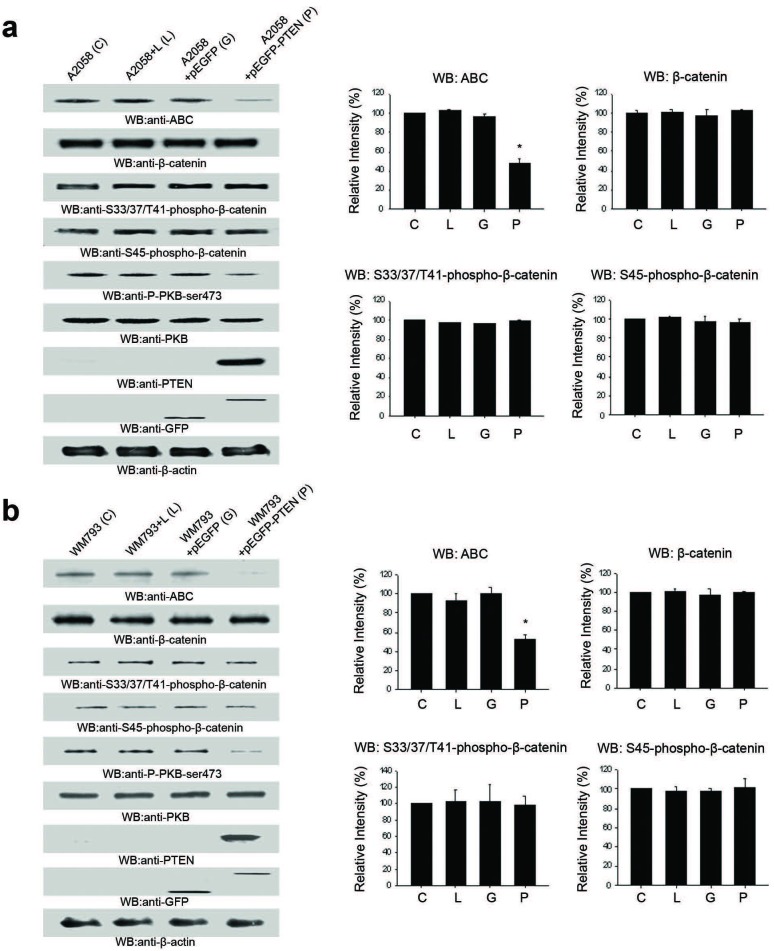

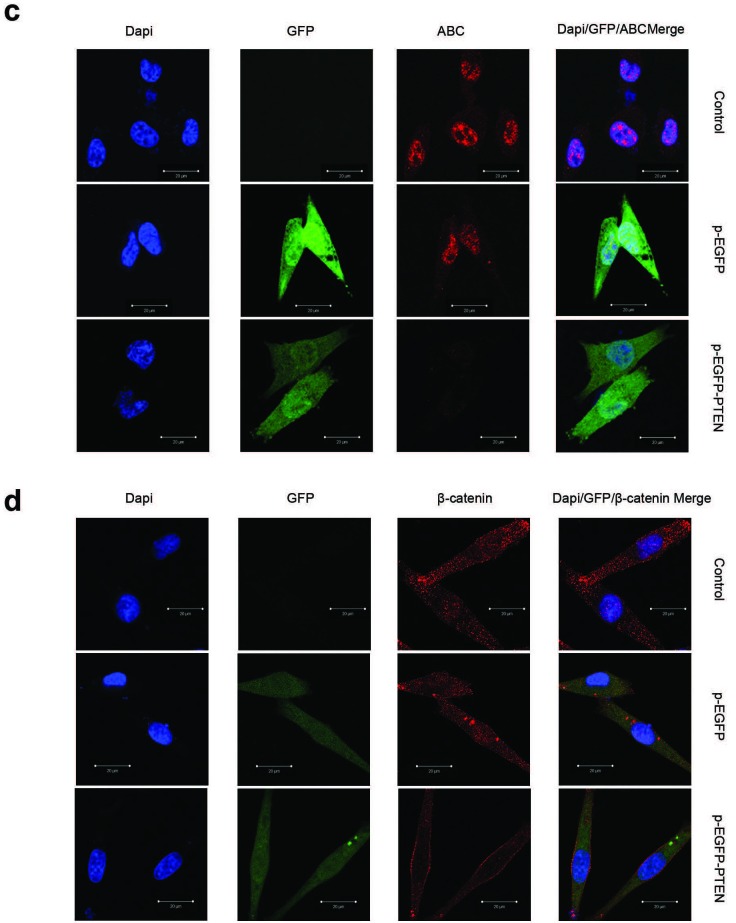
PTEN re-expression decreases ABC protein levels in PTEN null A2058 cells **A.** PTEN transfected A2058 cells show decreased ABC protein levels. PKB-Ser473-P levels decreased and total PKB levels remained unchanged. There was no alteration observed in the cellular levels of β-Catenin, phospho-S33, 37/T4-β-Catenin or phosphor-S45-β-Catenin under any conditions. **B.** A similar profile for ABC, β-Catenin, phospho-S33, 37/T4-β-Catenin and phospho-S45-β-Catenin was observed with PTEN re-expression in the PTEN-null WM793 melanoma cell line. Histograms are representative of densitometric analysis of 4 or more experiments and are reported as percentage of the respective control that is set at 100%. *p<0.05. **C.** Immunofluorescence analysis shows significant reduction of nuclear ABC (red) following PTEN re-expression (green) in PTEN-null A2058 melanoma cells. **D.** Re-expression of PTEN results in decreased localization of β-Catenin (Red) in nuclear and cytosolic compartments levels and increased localization to cellular membrane.

ABC levels decreased upon reintroduction of pEGFP-PTEN into A2058 cells (Figure 2A). However, there was no significant change in total β-catenin or phosphorylated β-catenin (Ser33/37, Thr41 and Ser45) levels (Figure 2A). Transfection with pEGFP-PTEN decreased P-PKB-Ser473 levels while total PKB levels remained constant. Similar results were observed with transfection of pEGFP-PTEN into the PTEN-null WM793B cells (Figure 2B).

Alterations in cellular levels and subcellular localization of β-catenin and ABC were further investigated with IF analysis. As shown in Figure 2C, A2058 cells express high levels of ABC that is mainly localized at the nucleus. ABC levels/localization were unaltered upon expression of p-EGFP (empty vector) in the cells. However, nuclear levels of ABC were significantly decreased upon expression of p-EGFP-PTEN. β-catenin is also expressed in high levels and dispersed throughout the cytoplasm and nuclei of A2058 cells without any focused localization at the plasma membrane (Figure 2D). This distribution of β-catenin was unaltered upon expression of pEGFP (Figure 2D). However, expression of pEGFP-PTEN resulted in a dramatic re-localization of β-catenin from the cytoplasmic and nuclear compartments to become primarily localized at the plasma membrane.

Collectively, these results show that the PI3K/PTEN pathway plays a role in regulating cellular/nuclear levels of ABC in cells. The results also indicate that although the PI3K/PTEN pathway does not regulate cellular β-catenin levels, it does regulate the subcellular localization of β-catenin.

### Inhibition of PI3K with Wortmannin decreases ABC levels

**Figure 3:**
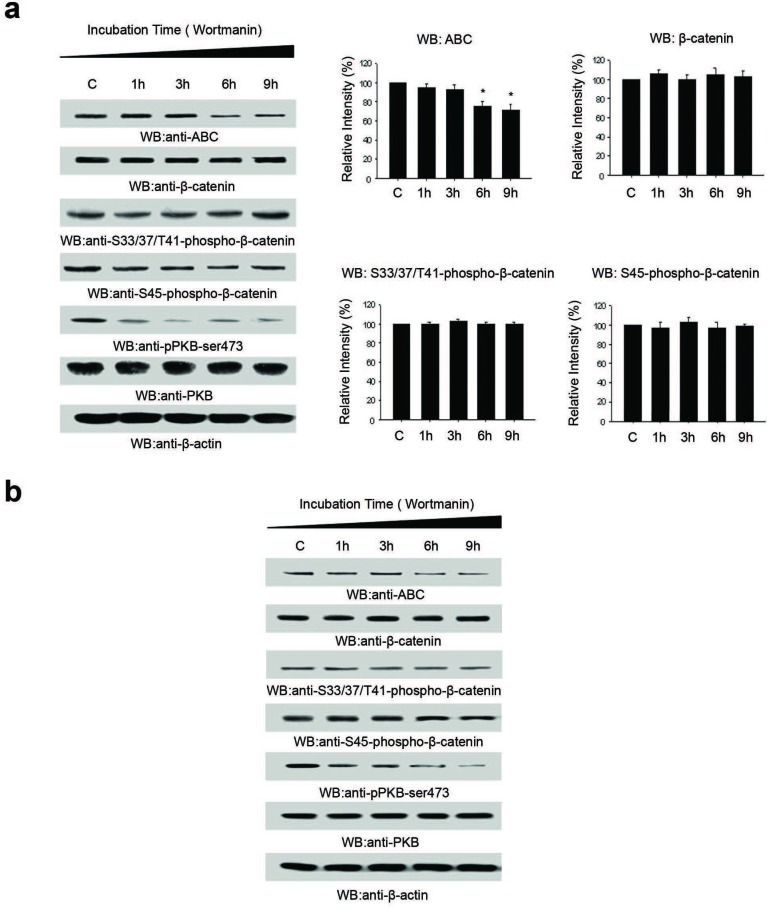
Inhibition of PI3K with Wortmannin decreases ABC levels independently of β-catenin **A.** Treatment of PTEN-null A2058 cells with Wortmannin resulted in decreasing cellular levels of ABC. However, there was no alteration in cellular levels of β-Catenin, phospho-S33, 37/T4-β-Catenin and phospho-S45-β-Catenin with Wortmannin treatment. **B.** A similar profile for ABC, β-Catenin, phospho-S33, 37/T4-β-Catenin and phospho-S45-β-Catenin was observed with Wortmannin treatment PTEN-null WM793 melanoma cell line. Histograms are representative of densitometric analysis of 4 or more experiments and are reported as percentage of the respective control that is set at 100%. *p < 0.05.

To further assess involvement of the PI3K pathway in regulating ABC levels, we treated A2058 cells with PI3K inhibitor, Wortmannin. As shown in Figure 3A, ABC levels decreased significantly after 6 hours of Wortmannin (2nM) treatment. Levels of P-PKB-Ser473 decreased within one hour of Wortmannin treatment with no change in the levels of PKB. β-catenin levels were unaltered as were the levels of Ser45- and Ser-33/37/Thr41-phosphorylated forms of β-catenin. Similar results were observed when WM793B cells were treated with Wortmannin (Figure 3B).

### ABC levels are not regulated by the Wnt pathway

**Figure 4:**
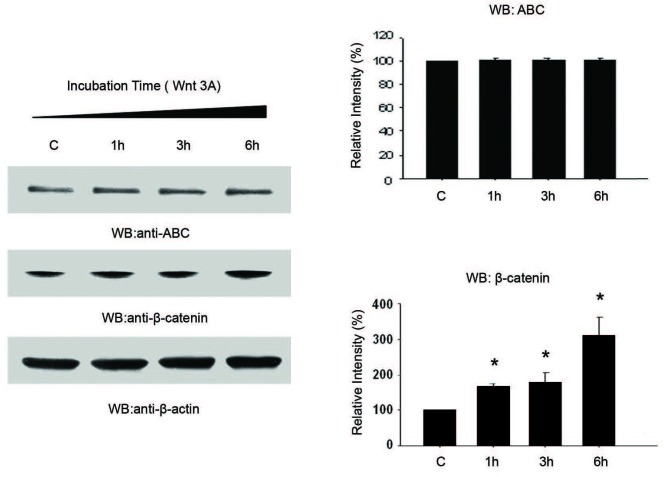
Wnt 3A alters cellular levels of β-catenin independent of ABC Treatment of A2058 cells with recombinant Wnt3A protein (100 ng/ml) resulted in increased cellular levels of β-catenin within 1 hour of treatment. There was no change in cellular levels of ABC with Wnt3A treatment. Histograms are representative of densitometric analysis of 4 or more experiments and are reported as percentage of the respective control that is set at 100%. *p < 0.05.

To investigate the contribution of the Wnt pathway in modulating ABC levels, we treated A2058 cells with purified recombinant Wnt-3a protein (100ng/ml). Activation of the Wnt pathway resulted in an increase in total β-catenin levels within 1hour of treatment (Figure 4). Interestingly, levels of ABC were stable for the total treatment period (Figure 4).

Collectively, the results from Figures 3 and 4 suggest that while total β-catenin is regulated by the canonical Wnt signaling pathway, ABC is regulated, at least partially, by the PI3K pathway and not the Wnt signaling pathway.

### Re-expression of PTEN-wild-type in A2058 cells downregulates β-catenin/TCF transcriptional activity

**Figure 5:**
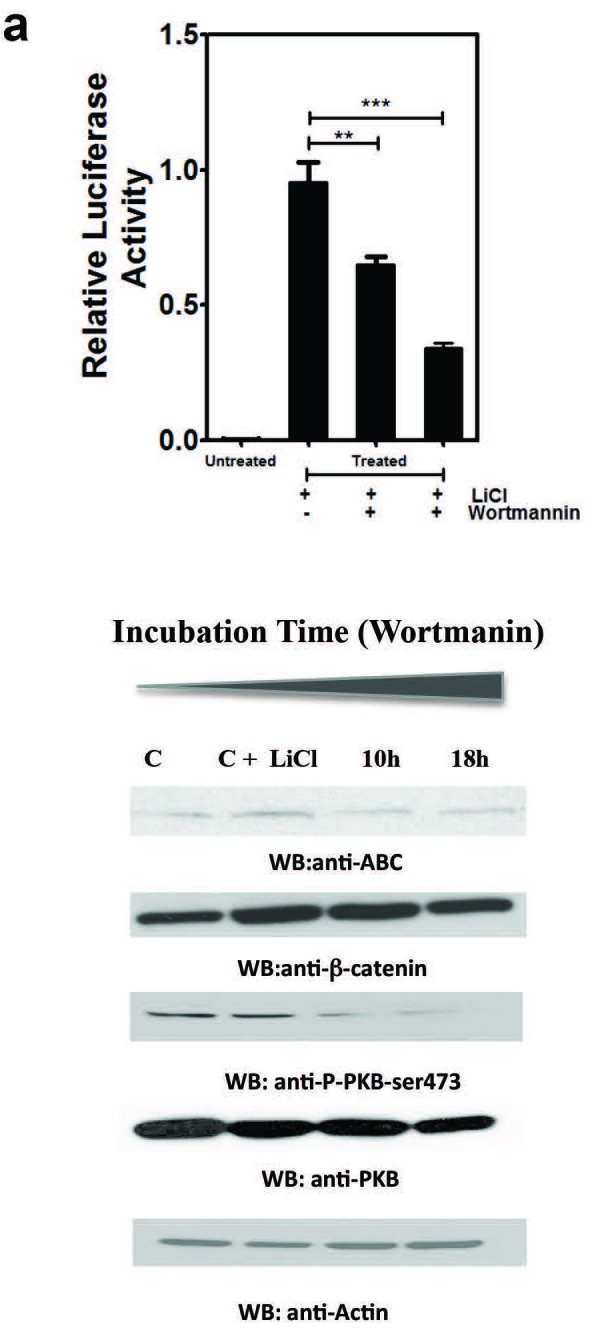

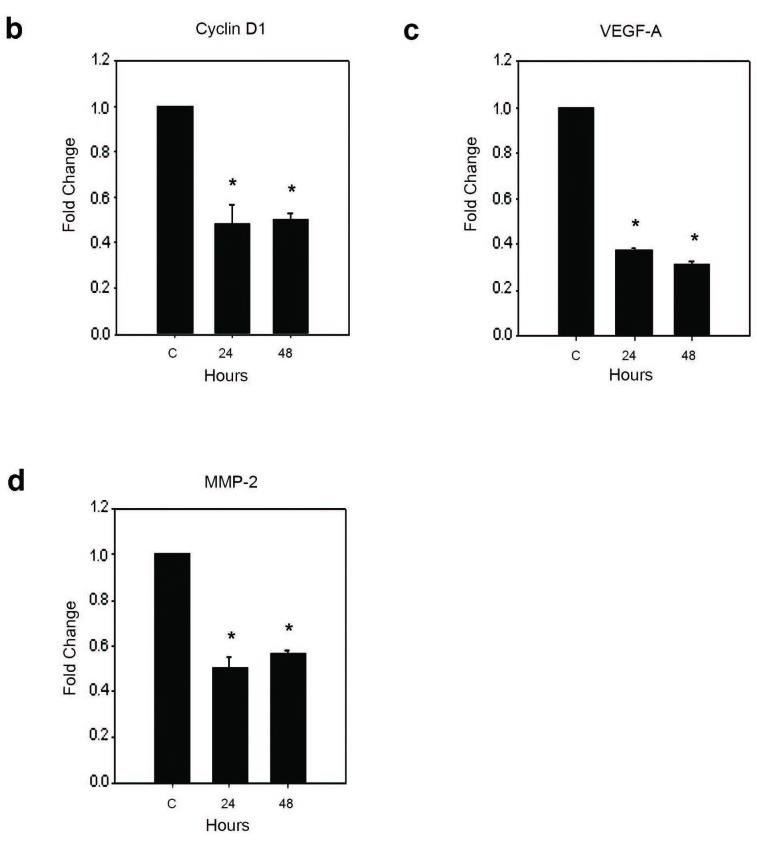
Inhibition of PI-3 Kinase pathway decreases ABC levels and transcriptional activity of Wnt/β-catenin pathway **A.** HEK 293 cells stably expressing TopFlash-Luciferase reporter (HEK293T) shows amplified TopFlash luciferase activity upon treatment with LiCl. This activity is severely attenuated in the presence of PI3 Kinase inhibitor Wortmannin. Panel below shows cellular levels of ABC, β-Catenin, PKB-473-P and PKB in HEK 293T cells under the same conditions are shown by Western blot analysis. A dramatic decrease in ABC, and not β-catenin levels, is noted with Wortmannin treatment. **B**.-**D.** PTEN re-expression in A2058 cells decreases mRNA expression of Wnt/β-catenin target genes, (B) Cyclin D1, (C) VEGF-A and (D) MMP-2. mRNA expression was measured by RT-qPCR at 24-48 hours following transfection in A2058 cells. Each group of data is representative of 4 or more experiments. *p < 0.05.

Since our results indicate that ABC levels are regulated by the PI3K pathway, we studied the effect of inhibiting the PI3K pathway on β-catenin/TCF transcriptional activity using the TOPFlash-Luciferase reporter (Figure 5A). We used HEK 293 cells stably transfected with pTA-TCF/LEF-luciferase reporter vector (HEK 293T) (Signosis). HEK 293T cells were treated with 10mM LiCl in the presence or absence of Wortmannin. TOPFlash activity was robustly induced upon treatment with LiCl in the absence of Wortmannin (control) (Figure 5A). However, the LiCl-induced TOPFlash activity was significantly attenuated in the presence of Wortmannin treatment for 10 and 18 hours (Figure 5A). Both β-catenin and ABC levels were increased upon treatment of the HEK293T cells with LiCl. However, only cellular levels of ABC, not β-catenin, were significantly decreased with Wortmannin treatment.

We next studied the effect of inhibiting the PI3K/PTEN pathway on the mRNA expression of selected β-catenin/TCF regulated target genes, including cyclin D1 (Figure 5B), VEGF-A (Figure 5C) and MMP-2 (Figure 5D) using RT-qPCR. GAPDH was used as an endogenous control. mRNA expression of all three genes were significantly decreased upon re-expression of pEGFP-PTEN in A2058 cells when compared to vector control.

### Re-expression of PTEN-WT in A2058 cells down-regulates PP2A phosphatase protein levels and activity

**Figure 6:**
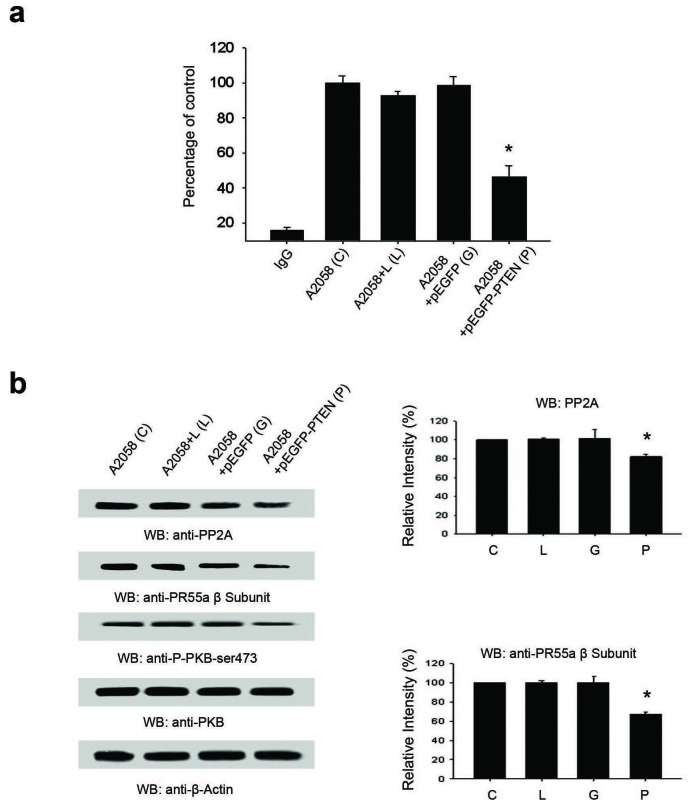
Cellular levels and activity of PP2A phosphatase is altered by re-expression of PTEN in A2058 cells **A.** The phosphatase activity of PP2A using the synthetic phosphopeptide K-R-pT-I-R-R was decreased significantly upon re-expression of PTEN in PTEN-null A2058 cells. Each group of data is representative of 4 or more experiments. **B.** There was also a significant decrease in the cellular levels of both PP2A, and more specifically, the level of the PR55α B subunit with PTEN re-expression in PTEN-null A2058 cells. Histograms are representative of densitometric analysis of 4 or more experiments and are reported as percentage of the respective control that is set at 100%. *p < 0.05.

Since the generation of ABC requires partial dephosphorylation of β-catenin, our next aim was to elucidate the identity of the phosphatase involved in this pathway. PP2A has been previously suggested as a regulator in the generation of ABC [8, 9]. We measured PP2A activity in A2058 cells in the presence or absence of expression of pEGFP-PTEN. A2058 cells exhibited high baseline PP2A activity which was significantly decreased upon pEGFP-PTEN re-expression (Figure 6A). Moreover, the high baseline PP2A activity in A2058 cells corresponded with high protein levels of PP2A and the regulatory PR55α B subunit (Figure 6B). Levels of PP2A and PR55α B were significantly decreased upon pEGFP-PTEN re-expression (Figure 6B).

### Inhibition of PP2A activity with okadaic acid decreases cellular ABC levels

**Figure 7:**
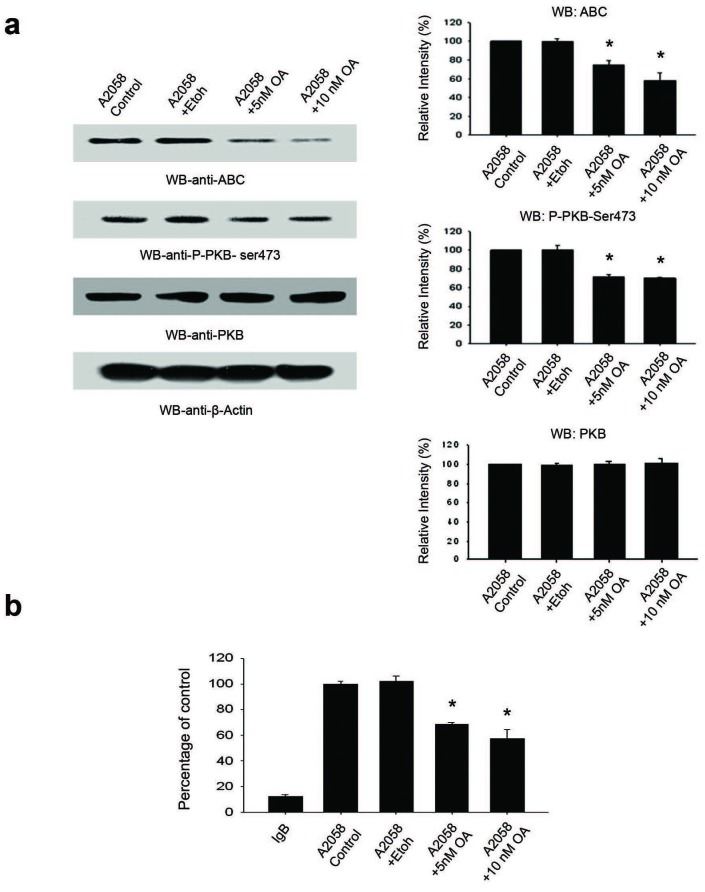
Inhibition of PP2A activity with okadaic acid decreases cellular ABC levels **A.** Treatment of A2058 cells with 5nM OA resulted in a significant decrease in ABC levels. ABC levels decreased further upon treatment with 10nM OA. Histograms are representative of densitometric analysis of 4 or more experiments and are reported as percentage of the respective control that is set at 100%. **B.** PP2A activity is significantly inhibited by the treatment of A2058 cells with 5nM and 10nM OA. Each group of data is representative of 4 or more experiments. *p < 0.05.

Since our data suggest that both ABC protein level and PP2A activity are regulated by the PTEN/PI3K pathway, we explored the putative role of PP2A in regulating ABC levels in the cell. A2058 cells were treated with or without okadaic Acid (OA) to inhibit PP2A activity and ABC levels were evaluated by Western Blot. Treatment of A2058 cells with OA (5nM and 10nM) resulted in a significant decrease in ABC levels (Figure 7A). Importantly, PP2A activity was significantly inhibited by the treatment of A2058 cells with 5nM and 10nM OA (Figure 7B).

## DISCUSSION

β-catenin, the central player of the canonical Wnt signaling pathway, functions as a potent oncoprotein that promotes malignant transformation, increases migratory capacity and invasiveness. The PI3K pathway has been identified as a metabolic signaling pathway that is integral to driving the malignant progression of many tumors. Here, we characterize a link between these two pathways in the regulation of the transcriptionally active form of β-catenin, ABC. We show a dramatic reciprocal relationship between PTEN and ABC in melanoma cells where loss/inactivation of PTEN is accompanied by a marked elevation in levels of ABC (Figure 1A). We further demonstrate this pattern in paired PTEN-null and PTEN-expressing prostate and breast cancer cells (Figure 1B), suggesting that this pattern of alterations is conserved between tumor-derived cell lines. Our results suggest that dephosphorylation of β-catenin to generate ABC is catalyzed by the phosphatase PP2A, which in turn is regulated by the PI3K pathway. Furthermore, our results demonstrate that induction of cellular ABC levels correlates with decreased membrane-localized β-catenin in a PI3K/PTEN-dependent manner. Hence, modification of β-catenin downstream of the PI3K pathway may regulate subcellular relocalization of β-catenin from its interactions at the adherens junction and induce a switch to β-catenin's role as a transcription factor.

We investigated the putative regulation of β-catenin by the PI3K pathway by inhibiting PI3K signaling in PTEN-null melanoma cell lines via two methods: (i) re-introduction of PTEN, and (ii) inhibition of PI3K signaling using an established inhibitor of PI3K. Taken together, we noted decreased cellular/nuclear ABC levels independent of changes in total cellular β-catenin levels (Figures 2A, 2B, 3A, 3B). Further, we observed a significant decrease in TOPFlash activity with Wortmannin treatment and decreases in expression of Wnt target genes cyclin-D1, VEGF-A and MMP-2 upon re-introduction of PTEN. This data suggests reduced transcriptional activity of β-catenin, likely as a result of reduced ABC levels in the nucleus with inhibition of PI3K signaling.

To investigate whether the Wnt pathway plays a role ABC regulation, we treated cells with Wnt3A, a canonical Wnt ligand that binds Frizzled (Fz) and consequently disables the destruction complex. Classically, activation of the canonical Wnt pathway has been associated with accumulation of β-catenin and a corresponding increase in nuclear β-catenin transcriptional activity. We observed that while treatment with Wnt 3A resulted in increasing levels of total cellular β-catenin, there was no identifiable change in levels of ABC (Figure 4). Collectively, these results suggest a novel model of β-catenin regulation where canonical Wnt signaling regulates β-catenin cellular stability but not its transcriptional activity, the latter being regulated by the PI3K pathway.

At first glance, our findings are partly discordant with data published by Staal et al. [7], whose group showed that canonical Wnt signaling induced by Wnt1 is sufficient to induce increased levels of ABC. This discrepancy in our findings can be attributed to a variety of factors, including disruptions or relative heterogeneity of factors in the canonical Wnt pathway in melanoma. The cells used by Staal *et al.* were Chinese-hamster ovary derived (CHO) cells, which express very low levels of PTEN (with high PI3K pathway activity) [31] and a regulated Wnt axis. In A2058 cells both the PI3K and Wnt axes are deregulated. Thus the CHO cells [7] present a different cellular and molecular environment than our model. Therefore, if our hypothesis is correct, i.e. that PI3K rather than Wnt signaling is responsible for activation of β-catenin, then in the presence of a deregulated PI3K pathway with limited availability of β-catenin (CHO cells), activation of Wnt signaling would increase β-catenin levels and thereby increase expression of ABC as reported by Staal et al.[7]. Conversely, in A2058 cells (our model) where β-catenin is in excess and the PI3K pathway is deregulated, stimulation of Wnt signaling would have little effect on ABC levels (Figure 4). In other words, if the limiting factor is substrate availability then stimulation of the Wnt pathway to increase β-catenin levels should result in higher levels of ABC. However, Wnt-stimulation would not alter ABC generation when β-catenin is already in abundance. Alternatively, there could be differences in the nature of the response elicited by Wnt3a compared to Wnt1 that may also account for our divergent results.

Several studies have previously explored connections/cross talk between the Wnt and PI3K pathways. A suggested intermediate in this cross talk is GSK-3β, a common component to both pathways. Sharma et al. [10] characterized GSK-3β-dependent crosstalk between the two pathways, showing that a mutation in one of β-catenin's N-terminal phosphorylation sites abolished the inhibitory effect of the PI3K inhibitor LY294002 on β-catenin-mediated androgen receptor augmentation (mutants used were S33F and S37A). This study implicated PI3K as a regulator of Wnt signaling. However, our study shows that the canonical Wnt pathway does not affect activation of β-catenin in the generation of ABC (Figure 4). Further, Sharma et al. [26] used mutants that abolished the ability of β-catenin to be fully phosphorylated at its N-terminus. The mechanisms underlying putative recognition of the β-catenin N terminus by PP2A have not been reported, but the absence of a normally phosphorylated N terminus might preclude PP2A from recognizing the mutant β-catenin as a target. Therefore the results presented by Sharma et al. cannot be taken as definitive evidence that GSK3β is indeed the locus for crosstalk between these two pathways. In addition, Sharma *et al.* used a β-catenin/AR co-transcriptional model of nuclear β-catenin activity, which may differ from the canonical β-catenin/TCF-4 mediated activity that we used as our model.

Previous studies have identified PP2A as a Ser/Thr phosphatase that may regulate the dephosphorylation of β-catenin to generate ABC [8]. PP2A and the related protein phosphatases PP4 and PP6 account for the majority of cellular Ser/Thr phosphatase activity [32]. The specificity of PP2A phosphatase activity is conferred by its assembly into a trimeric complex where the catalytic (C) subunit first dimerizes with a 65 kDa scaffolding subunit A. This core dimer can then associate through the A subunit with any of more than 16 regulatory B subunits. The regulatory B subunits determine substrate specificity and subcellular localization [33-37]. As an example, association with the PR55α B subunit facilitates activation of β-catenin while association with B56γ inhibits β-catenin transcriptional activity [9, 38]. Sablina et al. [9] showed a diverse mosaic of effects of PP2A regulatory subunit knockdown on β-catenin activity, with heterogeneity even within conserved B subunit families. Our results show that re-introduction of wild-type-PTEN in PTEN-null cells reduces PP2A and PR55α subunit expression and concordantly decreases PP2A activity, leading to a decrease in ABC levels. Our results therefore imply a dominance of PR55α-specific PP2A activity in the regulation of ABC. As such, in the context of proficient PTEN activity, suppression of PR55α-specific PP2A activity may also enable PP2A to take on an alternate role, as demonstrated by Sablina and colleagues, in the repression of β-catenin signaling, which is in accordance with its classically identified role as a tumor suppressor [39, 40]. Others such as Wu et al. have shown a tumor suppressive role of PP2A inactivation in pancreatic cancer cells correlated with decreased activation of β-catenin, which is concordant with our data [41].

There are a number of mechanisms by which PI3K signaling could influence PP2A signaling, both in terms of activation and in role specification by subunit composition. PP2A and PKB have been shown to interact both directly and indirectly: PP2A can dephosphorylate PKB [42] or may additively contribute to PKB activation [43], while PKB induces PP2A activity during normal development [44]. This heterogeneity is likely mediated through the mosaic of regulatory subunits and extrinsic factors that can influence PP2A specificity, such as TIPRL [45]. Our results support that the subunit PR55α is active in the dephosphorylation of β-catenin to generate ABC. This same subunit, PR55α, has been shown to selectively increase activity of PP2A against PKB [46], particularly at Thr308. In a normal setting, PKB may activate PP2A as a self-regulatory mechanism. However, in the setting of cancer, this feedback is not possible. A mutation in PKB or an interaction of another protein with PP2A or PKB could abrogate the effect of PP2A on PKB. Alternatively, the activity of PP2A on PKB could proceed normally without having any effect, in the background of a deregulated PI3K pathway inducing constitutive phosphorylation of PKB at Thr308 and Ser473. In this scenario, PP2A deregulation would represent a critical step in promoting progression in PTEN-null tumors. A potential indirect mechanism by which PKB may regulate PP2A activity is by regulation of mTORC1 [47], which itself has been shown to both positively [48] and negatively [49] influence PP2A activity, and may provide a complex mechanism for PKB-PP2A crosstalk downstream of PKB.

Collectively, our results support a heterogeneous role for PP2A in cancer and also a rather complex regulatory system underlying its activation. We predict that the PR55α B subunit of PP2A plays a dominant role in modulating PP2A activity in malignant melanoma and potentially in other cancers with dysfunctional or null PTEN, promoting an oncogenic role of PP2A as opposed to its classical tumor suppressor function. This model is supported both by the previous identification of PR55α as tumorigenic in other cancers [50], as well as demonstration of tumor suppression by other B subunits [51]. This switch in PP2A activity, from tumor suppressive to oncogenic, may be regulated by activation of PI3K signaling, and represent a crucial step in cancer progression via a regulation/increase in ABC activity.

**Figure 8:**
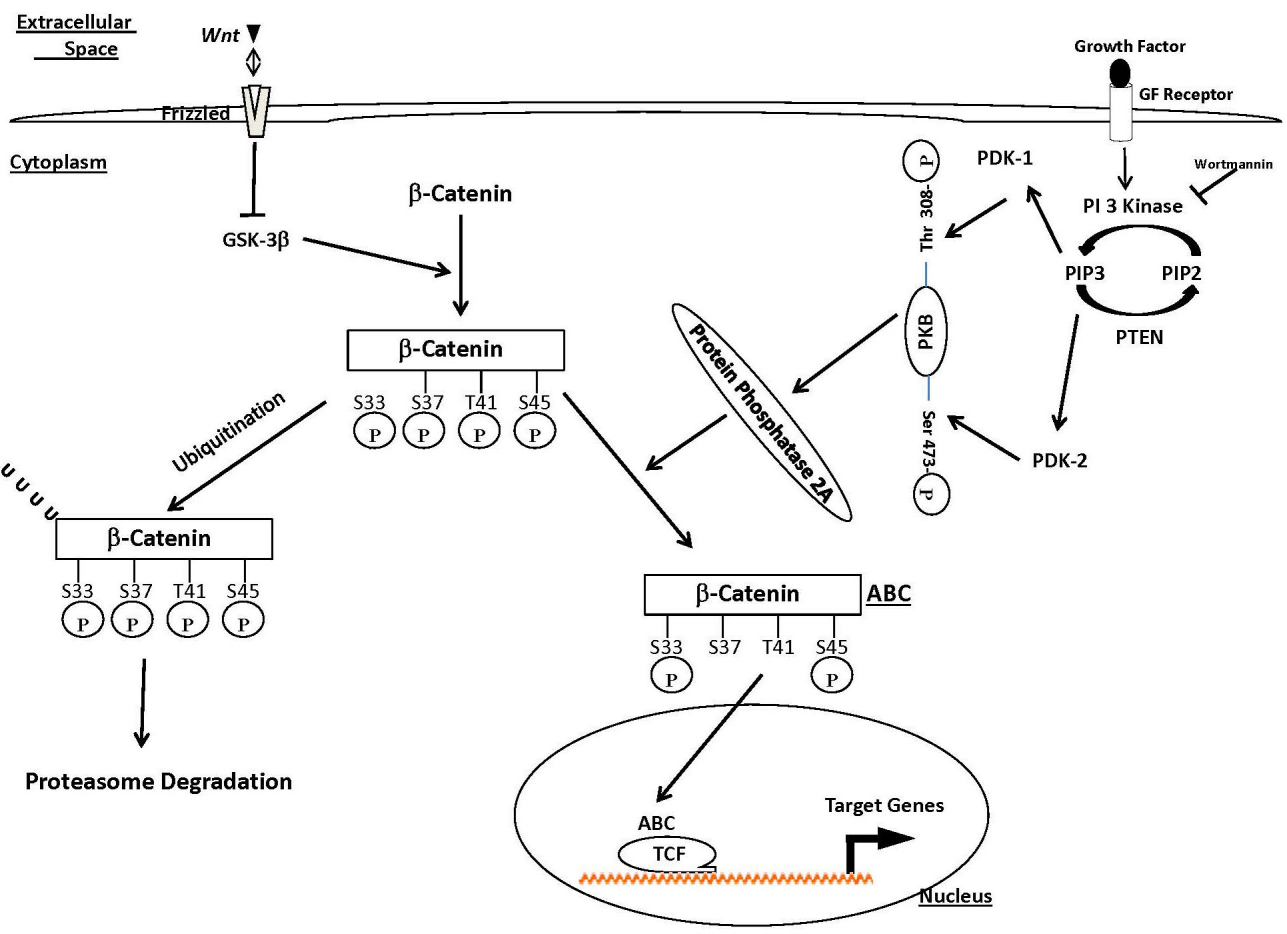
Schematic representation of mechanisms regulating β-catenin and ABC The cellular level of β-catenin is very tightly regulated by phosphorylation. Phosphorylation of β-catenin by glycogen synthase kinase-3beta (GSK-3β) results in its degradation. Prevention of phosphorylation upon activation of the Wnt pathway inhibits phosphorylation of β-catenin and stabilizes the protein which results in an increase of its levels inside cells. In this manuscript we show that while the canonical Wnt pathway stabilizes cellular levels of β-catenin it does not regulate cellular ABC levels. ABC levels are regulated by the PI3K pathway via activation of PP2A which then partially dephosphorylates β-catenin at serine 37 and threonine 41 to generate ABC and enable its transcriptional properties.

In summary, we have shown that the PI3K/PTEN pathway modulates β-catenin activation to form the transcriptionally active ABC in the setting of PTEN-null/inactive cell lines, while the canonical Wnt pathway regulates β-catenin stability. We also show, in agreement with work published by Bos et al. [8], a role for PP2A in the generation of ABC. Finally, we show that the PI3K/PTEN pathway regulates cellular ABC levels primarily through modulation of the PR55α B subunit, and that reintroduction of PTEN reduces activity of PP2A. These data imply a complex regulatory axis for β-catenin, whereby the canonical Wnt pathway stabilizes cellular levels of β-catenin but not ABC. ABC levels are regulated by the PI3K pathway via activation of PP2A, possibly in a manner that differs in subunit composition from traditionally tumor suppressive PP2A, which partially dephosphorylates β-catenin and enables its transcriptional properties (Figure 8). Therefore, we have elucidated an important node connecting two major oncogenic pathways which may be a viable target for the development of future novel therapeutics for blocking β-catenin signaling in the treatment of cancers where this pathway is deregulated.

## MATERIALS AND METHODS

### Cell lines and treatments

Human Epidermal Melanocytes (HeMa-Lp) (Cascade Biologics™) were grown in Medium 254, supplemented with 10% Human Melanocyte Growth Supplement, 10 μg/ml gentamicin and 0.25 μg/ml amphotericin B (Cascade Biologics™). Cell lines A375 A2058, DU145 and PC3 (ATCC) were grown in Dulbecco's Modified Eagle Medium (DMEM)/10% Fetal Bovine Serum (FBS). WM35, WM793B (Wistar line; ATCC), MCF7 and MDA-MB-468 were grown in Hyclone's RPMI-1640 medium/5% FBS. HEK 293 cells that were stably transfected with pTA-TCF/LEF-luciferase reporter vector containing 6 repeats of TCF/LEF binding sites, a minimal promoter upstream of the firefly luciferase coding region (Signosis). HEK 293T were grown in Dulbecco's Modified Eagle Medium (DMEM)/10% FBS. All cells were incubated at 37°C and 5% CO2. Where indicated, cells were treated with 2nM Wortmannin (Sigma) or 100 ng/ml purified recombinant human Wnt protein derived from CHO cells (R & D Systems). For okadaic acid treatment, cells were serum starved overnight followed by treatment with 5nM or 10nM okadaic acid in serum-free media for 2 hours.

### Plasmids and transfections

Cell lines were grown to 50% confluence and transfected with pEGFP-C3-hPTEN or pEGFP-C3 vector control (4 μg). All transfections utilized Lipofectamine2000 (Invitrogen) according to the manufacturer's protocols. Transfected cells were analyzed by Western blot, IF or quantitative RT-PCR.

### Whole cell lysis and nuclear extracts

Whole cell lysates were obtained by incubating with 100μl of lysis buffer (NP-40-DOC: 10mMTris-HCl pH 7.5, 1% NP-40, 0.5% Sodium Deoxycolate, 2 mM phenylmethylsulfonyl fluoride (PMSF), 80 ng/ml aprotinin, 40 ng/ml chymostatin, 40 ng/ml antipain, 40 ng/ml leupeptin, 40 ng/ml pepstatin) on ice for 10 minutes. Cellular debris was removed by centrifugation for 5 minutes at 16,000 x g. Nuclear and cytosolic extracts were separated using the NE-PER Nuclear Cytoplasmic Extraction Kit (ThermoScientific) according to the manufacturer's protocols. Protein concentrations were determined using Bicinchoninic (BCA) Protein Determination Assay (ThermoScientific).

### Luciferase assay for β-catenin/TCF promoter activities

We determined β-catenin/TCF-induced transcriptional activity using HEK 293T cells (Signosis). HEK 293T cells were treated with 10mM LiCl (for the activation of Wnt/β-catenin signaling) in the presence or absence of Wortmannin (2nM). Reporter activity was measured using a luminometer (Fluor Star OMEGA: BMG Labtech).

### Quantitative real-time PCR

Total RNA was isolated using RNeasy Kit (QIAGEN) as per the manufacturer's protocol. 1μg of total RNA was used for reverse transcription of Oligo (dT) (Invitrogen) and Superscript III reverse transcription (Invitrogen). Real time quantification of Cyclin D1, VEGF-A and MMP-2 were assessed using power SYBR Green PCR Master Mix (Applied Biosystems). GAPDH was used as the endogenous control. Samples were amplified with a precycling hold at 95°C for 15 seconds, 30 cycles of annealing and extension at 60°C for 1 minute. The following primers were used: Cyclin D1: sense (5`-CAT CTA CAC CGA CAA CTC CAT C-3`); Cyclin D1: anti-sense (5`-TCT GGC ATT TTG GAG AGG AAG-3`); VEGF-A: sense (5`-AGT CCA ACA TCA CCA TGC AG-3`); VEGF-A: anti-sense (5`-TTC CCT TTC CTC GAA CTG ATT T-3`); MMP-2: sense (5`-GGC CCT GTC ACT CCT GAG AT-3`); MMP-2: anti-sense (5`-GGC ATC CAG GTT ATG GGG GA-3`); GAPDH: sense (5`-TCAACGACCACTTTGTC AAGC TCA-3`); GAPDH: anti-sense (5`-GCTGGTGGTCCAGGG GTCTTACT-3`). Each measurement was performed in triplicate with Rotor-Gene-3000 instrument (Montreal Biotech Inc.) and analyzed using Rotor-Gene-6 Software. Gene expression was determined using the relative standard curve method normalized to GAPDH-binding protein expression. Histograms are reported as fold change of control which was set at 1.

### Immunocytochemistry

Cells were cultured on coverslips, washed with phosphate buffered saline (PBS) and fixed with 4% formaldehyde. Cells were permeabilized with 100% ice cold methanol at −20°C for 10 minutes and stained with primary antibody followed by treatment with Alexa Fluor^®^ 555-conjugated secondary antibody (Invitrogen) for visualization. Cells were visualized with IF imaging using a Carl Zeiss Laser Scanning Microscope and analyzed using LSM510 software.

### Immunoblotting

Cells were lysed with 50mM Tris buffer (pH 8.0) (150mM NaCl, 1% NP-40, 0.5% sodium deoxycholate, 1mM PMSF, 5μg/ml leupeptin and 25 μg/ml aprotinin). Protein concentration was quantified by BCA Assay. Proteins were separated by SDS polyacrylamide Gel (SDS PAGE) or Tricine Gel electrophoresis and transferred onto PVDF membranes (Millipore). PVDF membranes were blocked with 5% milk, probed with specific primary antibodies followed by peroxidase-conjugated secondary antibodies (GE Healthcare UK Limited) and visualized using Western Lightning^®^ Plus-ECL (PerkinElmer, LAS Inc.). Densitometric analysis normalized to the respective actin was performed by Image J software. Histograms are representative of four or more independent experiments and are reported as a percentage of control set at 100%. Statistical analysis was performed by Student's t-test (p<0.005) and Mann-Whitney Rank Sum test (p<0.05). Antibodies used: Antibodies to β-catenin, PKB, P-PKB-Ser-473, PTEN, S33/37/T41-phospho-β-catenin, S45-phospho-β-catenin, PP2A and PR55α (Cell Signaling), ABC (Millipore), GFP and Actin (Santa Cruz Biotech Inc.).

### PP2A activity assay

Cellular PP2A activity was assayed using a PP2A immunoprecipitation phosphatase assay kit (Upstate). Cells were washed in TBS, lysed with phosphatase assay buffer (20mM imidazole-HCl, 2mM EDTA, 2mM EGTA, 0.1% NP-40, pH 7.0, protease inhibitors). PP2A-C subunit was immunoprecipitated from total cell lysates (500 μg) using 4 μg of anti-PP2A-C antibody (clone 1D6, Upstate) and Protein A agarose for 2 h at 4°C. PP2A activity was assayed by incubating the immunoprecipitated protein with the synthetic phosphopeptide K-R-pT-I-R-R at 30°C for 10 min prior to detection with malachite green phosphate detection solution, according to the manufacturer's instructions. Phosphatase activity was normalized to the relative amount of immunoprecipitated C subunit using Western blot quantitation with Image J software.

## References

[1] Saito-Diaz K, Chen TW, Wang X, Thorne CA, Wallace HA, Page-McCaw A (2013). The way Wnt works: components and mechanism. Growth factors.

[2] Liu C, Li Y, Semenov M, Han C, Baeg GH, Tan Y (2002). Control of beta-catenin phosphorylation/degradation by a dual-kinase mechanism. Cell.

[3] Valenta T, Hausmann G, Basler K (2012). The many faces and functions of beta-catenin. The EMBO journal.

[4] Polakis P (2012). Wnt signaling in cancer. Cold Spring Harbor perspectives in biology.

[5] Jamieson C, Sharma M, Henderson BR (2012). Wnt signaling from membrane to nucleus: beta-catenin caught in a loop. The international journal of biochemistry & cell biology.

[6] Polakis P (1999). The oncogenic activation of beta-catenin. Current opinion in genetics & development.

[7] Staal FJ, Noort Mv M, Strous GJ, Clevers HC (2002). Wnt signals are transmitted through N-terminally dephosphorylated beta-catenin. EMBO reports.

[8] Bos CL, Kodach LL, van den Brink GR, Diks SH, van Santen MM, Richel DJ (2006). Effect of aspirin on the Wnt/beta-catenin pathway is mediated via protein phosphatase 2A. Oncogene.

[9] Sablina AA, Hector M, Colpaert N, Hahn WC (2010). Identification of PP2A complexes and pathways involved in cell transformation. Cancer Research.

[10] Seeling JM (1999). Regulation of beta-catenin signaling by the B56 subunit of protein phosphatase 2A. Science.

[11] Ratcliffe MJ, Itoh K, Sokol SY (2000). A positive role for the PP2A catalytic subunit in Wnt signal transduction. J Biol Chem.

[12] Lu Z (2003). Downregulation of caveolin-1 function by EGF leads to the loss of E-cadherin, increased transcriptional activity of beta-catenin, and enhanced tumor cell invasion. Cancer Cell.

[13] Ding Q, Xia W, Liu JC, Yang JY, Lee DF, Xia J, Bartholomeusz G, Li Y, Pan Y, Li Z, Bargou RC, Qin J, Lai CC, Tsai FJ, Tsai CH, Hung MC (2005). Erk associates with and primes GSK-3β for its inactivation resulting in upregulation of β-catenin. Mol Cell.

[14] Gosens R, Baarsma HA, Heijink IH, Oenema TA, Halayko AJ, Meurs H, Schmidt M (2009). De novo synthesis of β-catenin via H-Ras and MEK regulates airway smooth muscke growth. FASEB J.

[15] Hino S, Tanji C, Nakayama KI, Kikuchi A (2005). Phosphorylation of β-catenin by cyclic AMP-dependent protein kinase stabilizes β-catenin through inhibition of its ubiquitination. Mol Cell Biol.

[16] Fang D, Hawke D, Zheng Y, Xia Y, Meisenhelder J, Nika H, Mills GB, Kobayashi R, Hunter T, Lu Z (2007). phosphorylation of β-catenin by AKT promotes β-catenin transcriptional activity. J Biol Chem.

[17] Sears R, Nuckolls F, Haura E, Taya Y, Tamai K, Nevins JR (2000). Multiple Ras-dependent phosphorylation pathways regulate Myc protein stability. Genes Dev.

[18] Sansal I, Sellers WR (2004). The biology and clinical relevance of the PTEN tumor suppressor pathway. J Clin Oncol.

[19] Wu H, Goel V, Haluska FG (2003). PTEN signaling pathways in melanoma. Oncogene.

[20] Vivanco I, Sawyers CL (2002). The phosphatidylinositol 3-Kinase AKT pathway in human cancer. Nature reviews Cancer.

[21] Guldberg P, thor Straten P, Birck A (2007). Disruption of the MMAC1/PTEN gene by deletion or mutation is a frequent event in malignant melanoma. Cancer Research.

[22] Tsao H, Zhang X, Benoit E, Haluska FG (1998). Identification of PTEN/MMAC1 alterations in uncultured melanomas and melanoma cell lines. Oncogene.

[23] Trotman LC, Niki M, Dotan Z.A (2010). Cooperative interactions of PTEN deficiency and RAS activation in melanoma metastasis. Oncogene.

[24] Carracedo A, Pandolfi PP (2008). The PTEN-PI3K pathway: of feedbacks and cross-talks. Oncogene.

[25] Katoh M, Katoh M (2006). Cross-talk of WNT and FGF signaling pathways at GSK3beta to regulate beta-catenin and SNAIL signaling cascades. Cancer biology & therapy.

[26] Sharma M, Chuang WW, Sun Z (2002). Phosphatidylinositol 3-kinase/Akt stimulates androgen pathway through GSK3beta inhibition and nuclear beta-catenin accumulation. The Journal of biological chemistry.

[27] Naito AT, Akazawa H, Takano H, Minamino T, Nagai T, Aburatani H (2005). Phosphatidylinositol 3-kinase-Akt pathway plays a critical role in early cardiomyogenesis by regulating canonical Wnt signaling. Circulation research.

[28] Ding VW, Chen RH, McCormick F (2000). Differential regulation of glycogen synthase kinase 3beta by insulin and Wnt signaling. The Journal of biological chemistry.

[29] Ng SS, Mahmoudi T, Danenberg E, Bejaoui I, de Lau W, Korswagen HC (2009). Phosphatidylinositol 3-kinase signaling does not activate the wnt cascade. The Journal of biological chemistry.

[30] Hao L, Ha JR, Kuzel P, Garcia E, Persad S (2012). Cadherin switch from E- to N-cadherin in melanoma progression is regulated by PI3K/PTEN pahway through Twist and Snail. British Journal of Dermatology.

[31] Kerr F, Rickle A, Nayeem N, Brandner S, Cowburn RF, Lovestone S (2006). PTEN, a negative regulator of PI3 kinase signalling, alters tau phosphorylation in cells by mechanisms independent of GSK-3. FEBS letters.

[32] Janssens V, Goris J (2001). Protein phosphatase 2A: a highly regulated family of serine/threonine phosphatases implicated in cell growth and signalling. The Biochemical journal.

[33] Arroyo JD, Hahn WC (2005). Involvement of PP2A in viral and cellular transformation. Oncogene.

[34] Cohen PT, Brewis ND, Hughes V, Mann DJ (1990). Protein serine/threonine phosphatases; an expanding family. FEBS letters.

[35] Millward TA, Zolnierowicz S, Hemmings BA (1999). Regulation of protein kinase cascades by protein phosphatase 2A. Trends in biochemical sciences.

[36] Virshup DM (2000). Protein phosphatase 2A: a panoply of enzymes. Current opinion in cell biology.

[37] Xu Y, Xing Y, Chen Y, Chao Y, Lin Z, Fan E (2006). Structure of the protein phosphatase 2A holoenzyme. Cell.

[38] Zhang W, Yang J, Liu Y, Chen X, Yu T, Jia J (2009). PR55 alpha, a regulatory subunit of PP2A, specifically regulates PP2A-mediated beta-catenin dephosphorylation. The Journal of biological chemistry.

[39] Trotman LC, Alimonti A, Scaglioni PP, Koutcher JA, Cordon-Cardo C, Pandolfi PP (2006). Identification of a tumour suppressor network opposing nuclear Akt function. Nature.

[40] Bhardwaj A, Singh S, Srivastava SK, Arora S, Hyde SJ, Andrews J (2014). Restoration of PPP2CA expression reverses epithelial-to-mesenchymal transition and suppresses prostate tumour growth and metastasis in an orthotopic mouse model. British journal of cancer.

[41] Wu MY, Xie X, Xu ZK, Xie L, Chen Z, Shou LM (2014). PP2A inhibitors suppress migration and growth of PANC-1 pancreatic cancer cells through inhibition on the Wnt/beta-catenin pathway by phosphorylation and degradation of beta-catenin. Oncology reports.

[42] Kim KY, Baek A, Hwang JE, Choi YA, Jeong J, Lee MS (2009). Adiponectin-activated AMPK stimulates dephosphorylation of AKT through protein phosphatase 2A activation. Cancer research.

[43] Li Y, Wang X, Yue P, Tao H, Ramalingam SS, Owonikoko TK (2013). Protein phosphatase 2A and DNA-dependent protein kinase are involved in mediating rapamycin-induced Akt phosphorylation. The Journal of biological chemistry.

[44] O'Shaughnessy RF, Welti JC, Sully K, Byrne C (2009). Akt-dependent Pp2a activity is required for epidermal barrier formation during late embryonic development. Development.

[45] Nakashima A, Tanimura-Ito K, Oshiro N, Eguchi S, Miyamoto T, Momonami A (2013). A positive role of mammalian Tip41-like protein, TIPRL, in the amino-acid dependent mTORC1-signaling pathway through interaction with PP2A. FEBS letters.

[46] Kuo YC, Huang KY, Yang CH, Yang YS, Lee WY, Chiang CW (2008). Regulation of phosphorylation of Thr-308 of Akt, cell proliferation, and survival by the B55alpha regulatory subunit targeting of the protein phosphatase 2A holoenzyme to Akt. The Journal of biological chemistry.

[47] Inoki K, Li Y, Zhu T, Wu J, Guan KL (2002). TSC2 is phosphorylated and inhibited by Akt and suppresses mTOR signalling. Nature cell biology.

[48] Wang B, He Q, Mao Y, Chen Z, Jiang H, Chen J (2012). Rapamycin inhibiting Jurkat T cells viability through changing mRNA expression of serine/threonine protein phosphatase 2A. Transplant immunology.

[49] Liu L, Chen L, Luo Y, Chen W, Zhou H, Xu B (2010). Rapamycin inhibits IGF-1 stimulated cell motility through PP2A pathway. PloS one.

[50] Gilan O, Diesch J, Amalia M, Jastrzebski K, Chueh A (2015). PR55alpha-containing protein phosphatase 2A complexes promote cancer cell migration and invasion through regulation of AP-1 transcriptional activity. Oncogene.

[51] Nobumori Y, Shouse GP, Wu Y, Lee KJ, Shen B, Liu X (2013). B56gamma tumor-associated mutations provide new mechanisms for B56gamma-PP2A tumor suppressor activity. Molecular cancer research : MCR.

